# A standardised approach to pre-hospital RSI in the UK; utility, governance and content of current pre-induction checklists

**DOI:** 10.1186/1757-7241-23-S2-A16

**Published:** 2015-09-11

**Authors:** Mark R Burgess, Zane Perkins

**Affiliations:** 1 Department of Anaesthetics, Royal Cornwall Hospital, Cornwall, UK; 2 Centre for Trauma Science, Queen Mary University of London, UK

## Background

Pre-hospital Rapid Sequence Induction (RSI) is often performed on patients nearing physiological exhaustion in a complex, challenging environment [1]. Standard Operating Procedures (SOPs) and checklists can be used to improve patient safety [2]. The UK incidence of pre-hospital RSI and the utility and content of these safety constructs are unknown.

## Methods

A piloted survey was sent to the lead clinicians for all UK pre-hospital services with potential to be able to deliver RSI. Data was compared for high volume (>50 RSIs per annum) and low volume (≤50 RSIs per annum). Another piloted survey was sent to UK clinicians who themselves perform pre-hospital RSI. Current pre-induction checklists were compared and contrasted in terms of length, content and format.

## Results

58 individual services were identified with 76.8% responding. 69.8% of services have RSI capabilities, 26.7% of which throughout a 24-hour period. 1564 RSIs are performed per annum.

SOPs for RSI are used by 80% and checklists by 76.8% of services, (> commonly in high volume services). 40% of these teams have a separate ‘crash-induction’ checklist. Review and revision of checklist content with involvement of clinicians is more common in high volume Vs. low volume services. The majority of all clinicians surveyed responded that they both prefer a standardized approach to RSI and that it is safer than allowing absolute autonomy.

There was a large variation in length, content, style and format between the checklists analysed.

## Discussion

Despite the availability of pre-hospital RSI being sporadic, it is performed commonly in the UK. SOPs and safety checklists are used more commonly by high volume teams. In the challenging setting of pre-hospital care, these safety constructs may liberate spare bandwidth for utilization on other tasks, although care must be taken to limit the length and simplify the language as much as possible.
